# Conductance fluctuations in InAs quantum wells possibly driven by Zitterbewegung

**DOI:** 10.1038/s41598-017-06818-4

**Published:** 2017-08-11

**Authors:** Yu Iwasaki, Yoshiaki Hashimoto, Taketomo Nakamura, Shingo Katsumoto

**Affiliations:** Institute for Solid State Physics, University of Tokyo, 5-1-5 Kashiwanoha, Kashiwa, Chiba 277-8581 Japan

## Abstract

The highly successful Dirac equation predicts peculiar phenomena such as Klein tunnelling and Zitterbewegung (ZB) of electrons. From its conception by Erwin Schrödinger, ZB has been considered key in understanding relativistic quantum mechanics. However, observing the ZB of electrons has proved difficult, and instead various emulations of the phenomenon have been proposed producing several successes. Concerning charge transport in semiconductors and graphene, expectations were high but little has been reported. Here, we report a surprisingly large ZB effect on charge transport in a semiconductor nanostructure playing “flat pinball”. The setup is a narrow strip of InAs two-dimensional electron gas with strong Rashba spin–orbit coupling. Six quantum point contacts act as pinball pockets. In transiting between two contacts, ZB appears as a large reproducible conductance fluctuation that depends on the in-plane magnetic field. Numerical simulations successfully reproduced our experimental observations confirming that ZB causes this conductance fluctuation.

## Introduction

In 1930, Erwin Schrödinger^1^ found that the free relativistic particle described by the Dirac equation undergoes an oscillatory motion at the speed of light *c*. This phenomenon, called Zitterbewegung (from German, meaning “trembling motion”; and abbreviated to ZB), originates from the pure quantum nature of relativistic particles, for which the operator corresponding to its velocity does not commute with the Dirac Hamiltonian. This means the velocity, despite long being a most familiar quantity to physicists, is not a good quantum number for free relativistic particles. Although this remarkable nature stimulated much interest among researchers, its estimated amplitude *ħ*/*m*_0_ *c* ~ 386 fm, and angular frequency 2*m*_0_ *c* ^2^/*ħ* ~ 1.6 × 10^21^ rad/s for electrons, kept it out of experimental reach^2^. Successful ZB emulations have been achieved in entity models, including those with a single trapped ion^3^ and ultra-cold atoms in Bose–Einstein condensates^4, 5^. However, in more realistic emulations, a clear observation of ZB with electrons in artificial vacua, specifically solids^6–8^, has been a major open question. In two-dimensional electron systems with Rashba-type spin-orbit coupling (SOC)^9^, ZB is predicted to appear as a meandering of charge density^6^ with relatively large amplitude of more than ten nanometers^8^, which remains barely observable using nanoscale techniques and superfine structures. A possible observation was reported in a mesoscopic device^10^.

**Figure 1:**
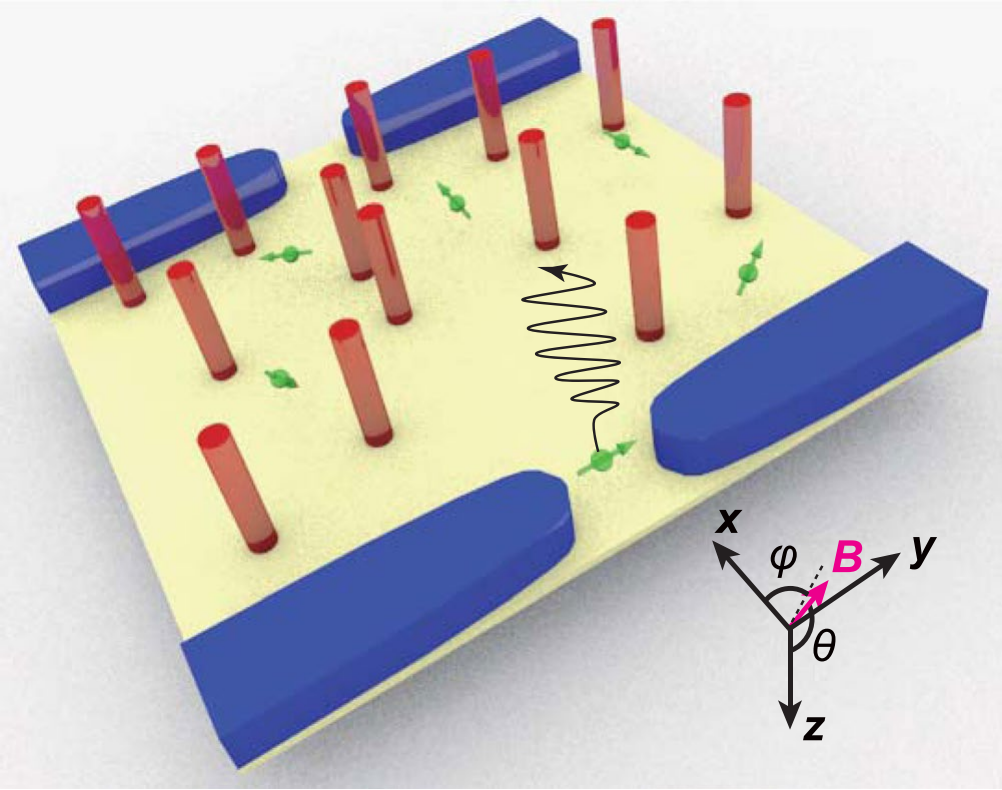
Pinball on a flat table. QPCs (blue) emit electrons (green balls) into a 2DEG (yellow) with impurities (red cylinders) scattering the electrons in the *x*-*y* plane. A 3D external magnetic field ***B*** is applied where azimuth *ϕ* and zenith *θ* are defined with respect to a *z*-axis set perpendicular to the *x*-*y* plane.

Here, we report the observation of ZB as a reproducible conductance fluctuation (CF) versus magnetic field in an InAs two-dimensional electron gas (2DEG) fabricated into an open billiard geometry with quantum point contacts (QPCs) as emitters and billiard pockets. However, the experiment resembles pinball played on a flat table (see Fig. 1) rather than as a billiard table, because the table has a considerable number of fixed scatterers (impurities). Using a numerical simulation, this “flat pinball” model was verified and demonstrated to exhibit meandering charge density with CF.

## Results

### ZB in Rashba model

Let us briefly view how ZB appears in the Rashba model described by the Hamiltonian

1 $$H=\frac{{{\boldsymbol{p}}}^{2}}{2{m}^{\ast }}+({\boldsymbol{a}}+{\boldsymbol{b}})\cdot {\boldsymbol{\sigma }},\quad \begin{array}{ccc}{\boldsymbol{a}}\equiv \frac{\alpha }{\hslash }(\begin{array}{c}{p}_{y}\\ -{p}_{x}\end{array}), & {\boldsymbol{b}}\equiv \frac{{g}^{\ast }}{2}{\mu }_{{\rm{B}}}(\begin{array}{c}{B}_{x}\\ {B}_{y}\end{array}), & {\boldsymbol{\sigma }}\equiv (\begin{array}{c}{\sigma }_{x}\\ {\sigma }_{y}\end{array}),\end{array}$$

where ***p*** is the electron momentum, *m* ^*^ the effective mass, *α* the Rashba SOC parameter, *g* ^*^ the effective Landé g-factor, *μ*_B_ the Bohr magneton, ${\boldsymbol{B}}\equiv {B}_{x}\hat{x}+{B}_{y}\hat{y}$ (with $\hat{x}$ and $\hat{y}$ as unit coordinate vectors) the in-plane magnetic flux density, and *σ*_*x,y*_ the Pauli matrices. As an initial condition (at *t* = 0), we consider a spin-up electron (the quantization axis is taken along *z*-axis) and a Gaussian wave packet centred on the origin with central wave vector ${\boldsymbol{k}}={k}_{0}{\hat{k}}_{x}$ (a vector of length *k*_0_ directed along the *k*_*x*_-axis). After an elapsed time *t*, the expectation value of the *y*-coordinate for finite |***a*** + ***b***| is^11^,

2 $$\langle y(t)\rangle =\frac{\alpha }{2}\frac{{({\boldsymbol{a}}+{\boldsymbol{b}})}_{y}}{{[\hslash \omega ({k}_{0})]}^{2}}\{1-\,\cos \,[\omega ({k}_{0})t]\}\equiv {y}_{0}\{1-\cos ({\omega }_{0}t)\},$$

where

3 $$\hslash \omega ({k}_{0})=|{\boldsymbol{a}}+{\boldsymbol{b}}|,$$

and the spin part of the wavefunction is written as (cos[*ω*(*k*_0_)*t*], sin[*ω*(*k*_0_)*t*]). The ZB amplitude in equation (2)vanishes in the limit *α* → 0 but approaches ${k}_{0}^{-1}$ for *α* → ∞.

**Figure 2:**
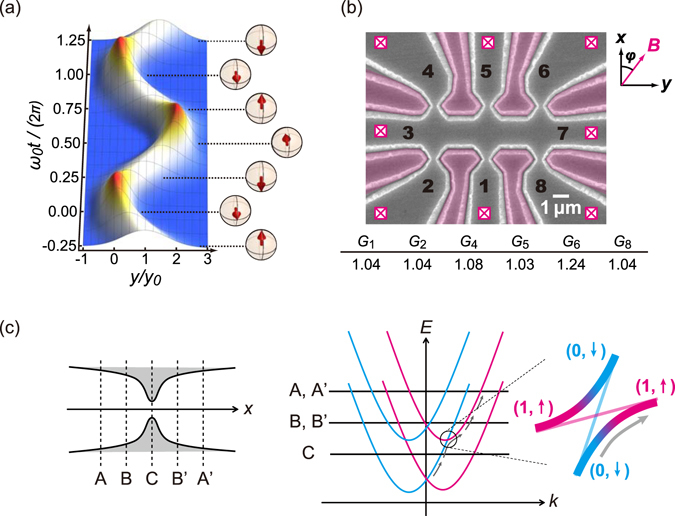
Experimental setup. (**a**) ZB in the Rashba model (for *ω*_0_ and *y*_0_, refer to equation (2). An electron wave packet, with spin parallel to the *z*-axis and an averaged momentum along *x* at time *t* = 0, oscillates along the *y*-axis in synchrony with the rotating spin in the *z*–*x* plane. (**b**) Scanning electron micrograph of the sample with terminals marked. The pink false-colour areas act as gates isolated by trenches to define the QPCs. Conductance of each QPC is listed below the micrograph in units of *G*_q_ (*T* = 55mK). (**c**) QPC diagram (left) and dispersion curves for the one-dimensional bands with a Rashba SOI (right). Red and blue curves indicate spin up and spin down, respectively. The magnification of a crossing point reveals a small avoided crossing. Within the spatial movement from C to B, an electron experiences the avoided crossing where the spin is flipped from down to up. A, C, and B indicate the positions of the Fermi energy during traversals—at the outlet, at the middle, and at a point between them, respectively^19^.

Figure 2(a) depicts the trace of a wave packet oscillating in the *y* direction according to equation (2)with its spin directions. As is easily understood, in a Rashba system, ZB appears as an electron orbit that meanders with a spin precession around the vector sum of ***B*** and an effective magnetic field originating from the SOC,

4 $${{\boldsymbol{B}}}_{{\rm{eff}}}=\frac{2}{{g}^{\ast }{\mu }_{{\rm{B}}}}{\boldsymbol{a}}=\frac{2\alpha }{{g}^{\ast }{\mu }_{{\rm{B}}}}(\begin{array}{c}{k}_{0}\\ 0\end{array}),$$

which is perpendicular to the momentum.

In the above formulae, the difficulties in the detection are apparent in that 1) the ZB amplitude is at most the Fermi wavelength, and 2) to attain this amplitude the ZB frequency in equation (3) should be very high—above 1 THz. We resolve these problems in the experimental setup introduced in the following.

### Experimental setup

To create a Rashba system, a 2DEG in a pseudomorphic InAs quantum well was grown by molecular beam epitaxy (see Supplementary Fig. 1 online). The layered structure has asymmetric (In, Ga)As/InAs interfaces in the well region, a structure that is commonly adopted for strong Rashba spin-orbit interaction (SOI)^12^. The electron mobility *μ* = 6.6×10^4^ cm^2^/Vs and the sheet carrier concentration *n* = 1.1 × 10^12^/cm^2^ were obtained from the longitudinal and Hall resistances. They produced a Fermi wavenumber of *k*_F_ = 2.6×10^8^ m^−1^, which corresponds to wavelength *λ*_F_ = 2.4 × 10^−8^ m. The effects of quantum confinement, wavefunction penetration into (In,Ga)As, and strain are renormalized as a shift in the in-plane effective mass in InAs from a bulk value of 0.023 *m*_0_ to 0.025 *m*_0_^13^. A Fermi velocity of *v*_F_ = 1.2 × 10^6^ m/s and a Fermi energy of *E*_F_ = 106 meV, *g* ^*^ = 8.6 ^14^ are then obtained. A value for the Rashba parameter *α* = 3.6 × 10^−11^ eVm is obtained (see Supplementary Fig. 2 online) from the amplitude modulation of the Shubnikov–de Haas (SdH) conductance oscillation^15^. These parameter values give an effective magnetic field strength in equation (4) of 38 T, and hence a ZB frequency of 4.6 THz.

The high-frequency problem can be resolved by fixing the ZB in a steady state using spin-polarized electrons. This can be accomplished with a quantum point contact (QPC) on a plateau of quantum conductance *G*_q_ ≡ 2*e* ^2^/*h* ^16–18^, which was predicted theoretically^19^ and confirmed experimentally^20^. Figure 2(c) illustrates the mechanism by which polarized spins of electrons pass through a QPC with Rashba SOI on conductance plateau *G*_q_. The right panel depicts the energy dispersion diagram of the 1D Rashba Hamiltonian. The lines A, B, and C, correspond to positions in the QPC are shown in the left panel. As an electron adiabatically passes through the QPC, its energy shifts sequentially from A → B → C → B’ → A’. During the transition from C to B’, the avoided crossing between (0, ↓) and (1, ↑) flips the electron spin. As a result of the “one-way spin rotation”, the spin polarization on plateau *G*_q_ reaches 0.7. Here, the spin separation of the dispersion branch comes from the *αp*_*x*_ *σ*_*y*_/*ħ* term, and the avoided crossing comes from −*αp*_*x*_ *σ*_*y*_/*ħ* in (1). The Rashba SOI is thus necessary for spin polarization.

Figure 2(b) shows a scanning electron micrograph of the sample with terminals marked. We take Cartesian coordinates as shown in the figure. The *z*-axis is perpendicular to the plane. The structure was fabricated by electron beam lithography and dry etching of trenches. The trench-gate technique is commonly used for InAs-based heterostructures^21^ as the Schottky gate technique is unsuitable. The structure consists of a main conducting strip (terminal 3–7) with a width of 2 μm and six QPCs opened on it. The main problem is that the gates (pink false-coloured regions) are common to adjacent QPCs and hence individual control of the QPC conductance is difficult. Fortunately, after much trial and error, we succeeded in tuning the QPC conductances at around 1.0 *G*_q_ with all gates grounded (except QPC-6); see Fig. 2(b). The fact that such a coarse tuning was successful indicates that the QPCs are on the 1.0 *G*_q_ plateau, where the conductance is less sensitive to the gate voltages than in other regions (see Supplementary Fig. 3 online).

Then, in a plot of the conduction through any combination of two QPCs, we emitted spin-polarized electrons into the main strip region in one QPC and collected them through the other one. Because of the electron-hole symmetry, this holds even for conduction paths with QPC-6, in which the polarization may be insufficient because *G*_6_ is slightly higher than 1.0 *G*_q_ (Fig. 2(b)). However, the effective opening of the QPCs is around 500 nm, which is still much wider than the maximum amplitude of ZB. Here, we attempted to solve the problem using scatterers in the strip as amplifiers of the meandering motion. Because the width of the present mean free path *l*_0_ ~ 1 μm is about half that of the main strip, the emitted electrons should experience a few scatterings before reaching a pocket. If we approximate such a scattering with a classical scattering for a hard cylindrical wall of radius *R*, the scattering angle *γ* given impact factor *b*_i_ is *γ* = 2cos^−1^(*b*_i_/*R*). As *b*_i_ oscillates via ZB with in-plane magnetic field ***B*** with amplitude Δ*b*_i_, the oscillation is amplified by *l*_0_Δ*b*_i_/*R*. The potential range *R* is roughly estimated to be an effective Bohr radius of 34 nm in InAs, which is even smaller than ${k}_{{\rm{F}}}^{-1}$. Hence, the amplified ZB can reach an order of *l*_0_ ~ 1 μm, which can be resolved with the present QPCs.

This rough classical sketch of scattering needs correcting in a quantum mechanical treatment, in that the spread of the wave packets weakens the amplification. However, as experimentally confirmed in scanning gate microscopy^22^, the wave packets emitted from a QPC travel for surprisingly long distances without smooth spreading because wave focusing occurs. Therefore, we can expect that semi-quantitatively the above classical analysis holds, and hence the experiment can be viewed as pinball played on a flat table with six pockets. The size of the ball (wave packet) may be larger than the pins (scattering centres), although minor wobbling in the orbits (*i.e*., ZB) is amplified by the scattering.

### Conductance fluctuations

**Figure 3:**
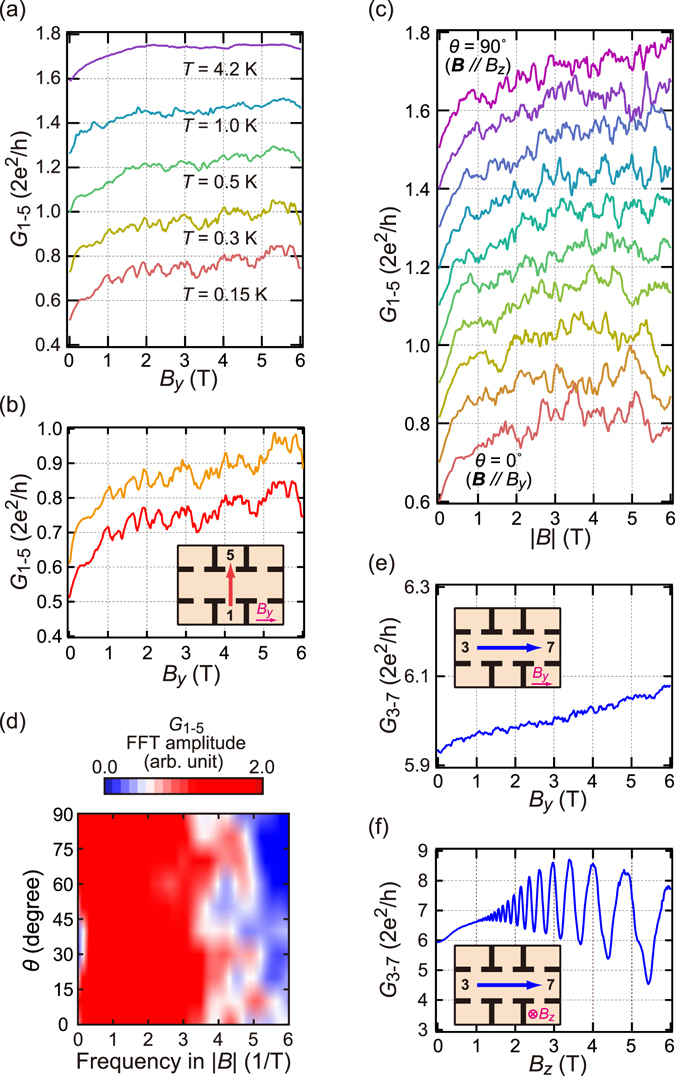
Reproducible conductance fluctuation versus magnetic field. (**a**) *G*_1–5_ as a function of *B*_*y*_ for different temperatures with offset 0.2 *G*_q_. (**b**) *G*_1–5_ for two individual magnetic sweeps with offset 0.1 *G*_q_. The inset shows the terminal configuration. (**c**) *G*_1–5_ at 70 mK versus field amplitude |*B*|. The direction of the magnetic field (the zenith angle *θ*) is rotated from *θ* = 0 to 90° at azimuth angle φ = 90°. The steps in the angle and conductance offsets are 10° and 0.2 *G*_q_, respectively. (**d**) 2D plot of the FFT amplitude spectrum of the conductance fluctuation versus |*B*| from 1.5 to 6 T and *θ*. (**e**) and (**f**) *G*_3–7_ at 70 mK (see inset for the configuration) plotted against *B*_*y*_ and *B*_*z*_, respectively.

Hereinafter, we denote the two-wire conductance between terminals *i* and *j* by *G*_*i*–*j*_. Figure 3(a) shows the temperature variation of the two-wire conductance *G*_1–5_ as a function of the in-plane magnetic field along the *y*-axis *B*_*y*_. As temperature decreases, aperiodic CFs with increasing *B*_*y*_ became visible and its amplitude increases. CFs are reproducible, that is, for two independent field sweeps at the same temperature, almost the same pattern appears [Fig. 3(b)]. Resemblances are also appreciable between patterns for different temperatures. CFs were observed for an arbitrary pair of electrodes with a QPC connection.

The result indicates that CFs are modulated by an external magnetic field similar to how they appear in the pinball model simulation. However, these features of CFs are also reminiscent of the so-called universal conductance fluctuations (UCF)^23^. We therefore checked whether this was possible. Because the applied field is in-plane, we can eliminate the quantum interference effect through random paths tuned via the Aharonov–Bohm (AB) phase. However, there still remains the possibility of interference in the spin part of the wavefunction, which is modified by a spin precession tuned via the in-plane magnetic field (spin-UCF)^24^; to eliminate this possibility, we performed the following two experiments.

The first is the rotation of the field direction from in-plane to perpendicular-to-plane (*i.e*., rotating the elevation angle from 0 to *π*/2). The result [Fig. 3(c)] shows there is no significant variation in the amplitude and frequency distributions resulting from pattern changes involving angle [Fig. 3(d)]. This is inconceivable for an interference-type CF because, if there is such a network of spatial interference and the perpendicular component of the field is increased, the AB phase modulation participates in the interference modulation introducing some qualitative and systematic change in CF that stems from the geometric nature of the AB phase. The second is the magnetic response of *G*_3–7_, *i.e*., transport without QPCs (and hence without spin polarization). Figure 3(e,f) displays the result, for which a considerable change is seen from Fig. 3(c), in that in *G*_3–7_ almost no fluctuation is seen in the in-plane field and an ordinary SdH oscillation appears in the perpendicular field (although the SOI modulation is reduced, probably resulting from loose confinement into the strip). This also indicates that the CFs are not caused by an interference because if such an interference occurred, it inevitably appears in *G*_3–7_, the path of which passes through the strip. Note that in the configuration of 1 to 5, the QPCs dominating the absolute value of the conductance are connected in series to the strip. Hence the difference in the average number of conductance channels does not explain the diminishing fluctuation in *G*_3–7_.

The source of the CFs is then the ZB modulation of scattering in the pinball system; therefore, we further checked the nature of CFs. The pinball model has two temperature factors; one is the spin polarization at QPCs and the other is the strength of the effective field ***B***_eff_. The latter is related to Rashba splitting, which is approximately 6 K in the present case. The CF is visible at temperatures of an order-of-magnitude lower. Hence, within the model, the temperature dependence observed [Fig. 3(b)] should come from spin polarization, which depends on the energy diagram inside the QPCs. A derivation of the explicit temperature dependence is difficult, although an order-of-magnitude estimation is reasonable because the effective *E*_F_ is lower by an order of magnitude inside the QPCs.

### Azimuth angle dependence

**Figure 4:**
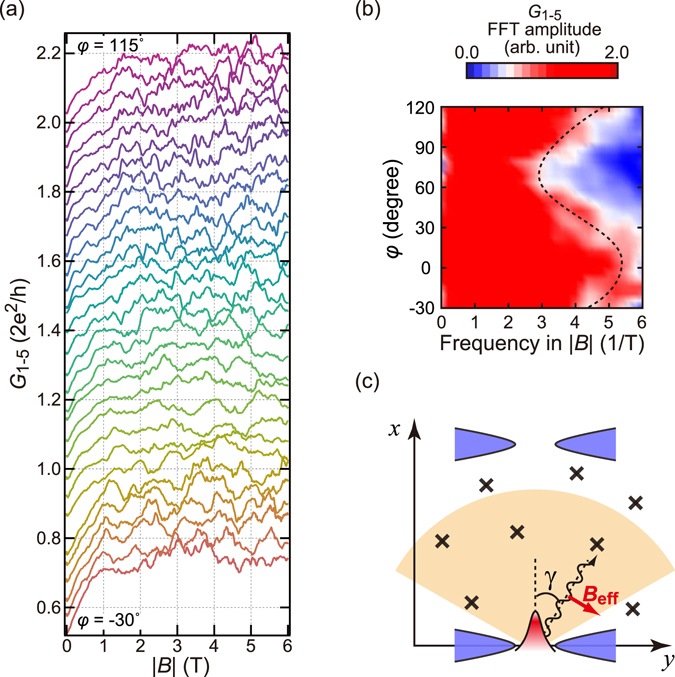
Effect of azimuth angle in the magnetic field on the conductance fluctuation. (**a**) Conductance fluctuation in *G*_1–5_ at 150 mK versus the magnetic field amplitude |*B*| for different azimuth angles *φ* from −30° (bottom) to 115° (top). The step in *φ* and the offset in *G*_1–5_ are 5° and 0.05 *G*_q_, respectively. (**b**) FFT amplitude spectrum of the conductance fluctuation plotted against |*B*| ranging from 1.5 to 6 T and *φ*. The broken line represents the smoothed *φ*-tendency given in equation (5). (**c**) Schematic of an electron path. Electrons transit over the fan-like beige area.

Figure 4(a) shows the variation of CF with azimuth angle φ for in-plane magnetic field. The fluctuation pattern changes with φ depending on its resemblance to neighbouring patterns. A fast-Fourier-transform (FFT) plot of the amplitudes versus φ (Fig. 4(b)) indicates some systematic angular dependence on spectral width.

Let us check the correspondence between the pinball model and the experimental results. First we consider electrons emitted from a QPC parallel to the *x*-axis. Their spins as well as ***B***_eff_ are parallel to the *y*-axis; the former is concluded from the theory in ref. 16 and the latter from equation (1). Because the spin and the effective field are parallel, there is neither a spin-precession nor a trembling motion.

Next we consider a path with an oblique angle *γ* to the *x*-axis [Fig. 4(c)]. The length of ***B***_eff_ + ***B*** is approximated as ${B}_{{\rm{eff}}}+B\,\cos \,\xi$ for $|{{\boldsymbol{B}}}_{{\rm{eff}}}|\gg |{\boldsymbol{B}}|$, where *B* is the external field strength and $\xi \equiv \pi \mathrm{/2}-\varphi +\gamma$ is the angle between ***B***_eff_ and ***B*** vectors. The “wavelength” of ZB is estimated as ${\lambda }_{{\rm{Z}}B}\equiv \pi {\hslash }^{2}/\alpha {m}^{\ast }\approx 270\,{\rm{nm}}$ and the maximum modification of ${\lambda }_{{\rm{Z}}B}$ with an external field for *B* = 6 T and $\xi =0$ is 34 nm. This means only a single cycle of meander changes in traversing 2 μm in a magnetic field of strength *B* = 6 T.

However, this does not mean maximum frequency 1/6 T^−1^ is expected in the CF. As noted the magnetic field change of 6 T on average corresponds to the change of single cycle in the ZB. The width of the strip is designed to be about twice the electron mean free path and many of the electrons traversing across it experience single scattering, which angularly amplifies the ZB. Hence *N* scatterings inside the fan of electron diffraction (the beige region in Fig. 4(c)), there should appear *N* to 2*N* such conductance peaks within the 6 T field, forming a fluctuation pattern. The density and sharpness of the conductance peaks increase for channels with more scatterings, whereas peak heights decrease. In the FFT power spectra, such gradual tailing to high frequencies is actually observed. In the conduction from terminal 5 to 1, the paths should distribute around *γ* = 0. Hence, we made the further approximation $B\,\cos \,\xi  \sim -B\,\cos \,\gamma \,\sin \,\varphi$. Moreover, the *φ*-dependence of the power spectral width *W*_p_ is roughly

5 $${W}_{{\rm{p}}}(\phi )\propto \frac{{l}_{0}}{{\lambda }_{{\rm{Z}}B}^{2}}|\frac{{\rm{\Delta }}{\lambda }_{{\rm{Z}}B}}{{\rm{\Delta }}B}|={l}_{0}\frac{\pi {g}^{\ast }{\mu }_{{\rm{B}}}}{{v}_{{\rm{F}}}}|\sin \,\phi |,$$

where *l*_0_ is the averaged ZB path length. Although the approximation is very coarse, equation (5) suggests that *W*_p_ takes a maximum at *φ* = 90° and a minimum at *φ* = 0°. In Fig. 4(b), the |*B*| frequency has a maximum at *φ* = 70° and a minimum at *φ* = 10°, which agrees qualitatively with equation (5).

**Figure 5:**
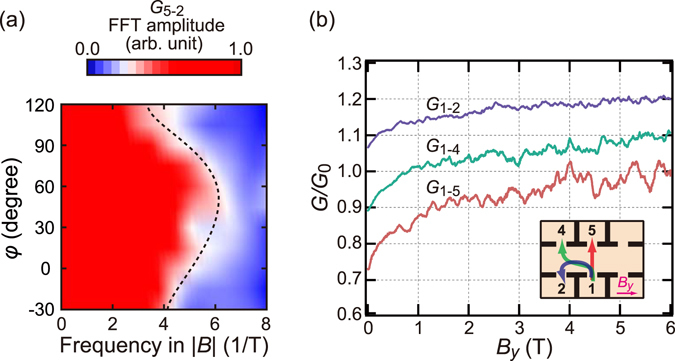
Effect of probe configuration. (**a**) The same FFT map as Fig. 4(b) but for *G*_5–2_ at *T* = 150 mK. (**b**) *G*_1–2_, *G*_1–4_, and *G*_1–5_ versus *B*_*y*_, for the configurations illustrated in the inset. The data are normalized by *G*_0_ = *G* (*B*_*y*_ = 6T) and displayed with offset 0.1 *G*_q_.

An interesting test is to change the combination of electrodes. Figure 5(a) shows results of the same experiment as Fig. 4(b) but for conduction between electrodes 5 and 2. Now, the centre of the path distribution is around *γ* = *π*/4, and the *φ*-dependence in equation (5) changes to |sin(*π*/4 + *φ*)| as is indeed observed in Fig. 5(a). Figure 5(b) shows the CFs for three different combinations of electrodes under an external magnetic field with *φ* = *π*/2. The most interesting is *G*_1–2_, in which backscattering is inevitable; hence, paths with multiple scatterings survive, resulting in a reduced amplitude fluctuation and higher frequencies. Similarly *G*_1–4_ is affected by multiple scatterings, although their proportion is not as large as *G*_1–2_.

### Tight binding calculation

Thus far, we have checked ZB in a simple pinball model from various viewpoints. Here, we show that ZB and the impurity scattering cause significant fluctuations in the conductance between QPCs in the Rashba model with numerical calculations.

**Figure 6:**
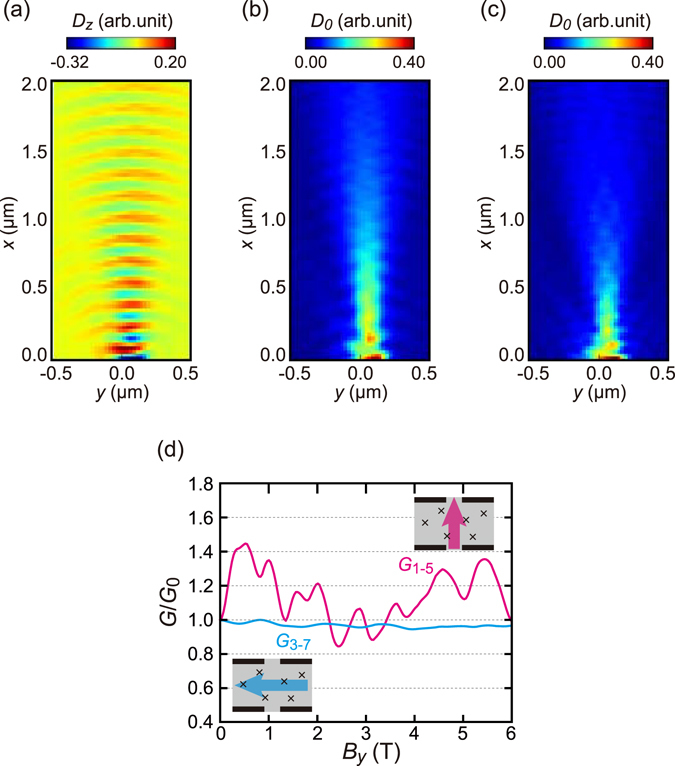
Numerical simulation with ZB reproducing the conductance fluctuation. (**a**) and (**b**) Calculated spatial distributions of the *z*-component of spin (*D*_*z*_) and the probability amplitude (*D*_0_), respectively, for a two-dimensional electron propagating from the emitter QPC at the origin under a zero-magnetic field. As boundary condition, the spin at the QPC is taken to be polarized along *x*; no scattering centre is introduced. (**c**) The same plot of *D*_0_ as (**b**), but with *B*_*y*_ = −3.5 T. (**d**) Calculated *G*_1–5_ (with polarization) and *G*_3–5_ (without polarization) as a function of *B*_*y*_. Random scattering centres are introduced. The data are normalized by *G*_0_ = *G*(*B* = 0). The probe configurations are illustrated in the insets.

Figure 6(a–c) shows the spatial distribution of spin-polarized electrons in a steady flow from a narrow constriction into a clean two-dimensional region. Clear meandering is observed, confirming the existence of ZB. Figure 6(d) plots the electron conductance with and without spin-polarization between two constrictions through a strip region with scatterers.

The conductance for spin-polarized electrons *G*_1–5_ shows a large aperiodic response with the amplitude of more than 0.1 *G*_0_ for an in-plane magnetic field ranging from 0 T to 6 T, whereas this CF is much smaller for unpolarized electrons *G*_3–7_. Note that we take an absorptive boundary condition, that is, the electrons are annihilated without reflection at the boundaries. The condition breaks unitarity while suppresses the unrealistically strong interference coming from the lack of phase decoherence in the calculation. The unitality is also not preserved in experiments as well because of the open geometry of the sample. The residual interference causes ordinary CF in *G*_3–7_ for spin-unpolarized electrons.

In the experiment, diffusive conductance constitutes offset conductance (0.5 *G*_q_ in Fig. 3(b) for example). Nevertheless, in the calculation, it is eliminated by the absorptive boundary, which explains why the simulated fluctuations are much larger (about 0.4*G*_0_) than the experimental fluctuations (about 0.1*G*_0_); see Fig. 6(d).

The result indicates that, in a Rashba system with scatterers, ZB may cause CF, providing evidence supporting the pinball model (see Supplementary Fig. 4 online). An alternative explanation of the results is a chaotic quantum or classical billiards effect. Measuring other samples with different impurities distribution would provide effective verification.

## Methods

### Two-dimensional electron system in an InAs quantum well

The substrate is an InAs quantum well, grown on a (001) InP using molecular beam epitaxy. From the bottom up, it consists of InAlAs and InGaAs (700 nm) as buffer layers, InAs (4 nm), InGaAs (4 nm), InAlAs (10 nm), n-InAlAs (40 nm), InAlAs (5 nm), and InGaAs (2 nm). QPCs were fabricated by electron-beam lithography and Ar dry-etching with a depth of 300 nm. Contacts were fabricated by AuGe deposition (100 nm) and subsequent annealing (280 degree, 5 min).

The specimen was cooled to 70 mK in a dilution fridge with a superconducting solenoid supplying the magnetic field. A conventional lock-in technique was used for measuring two-wire conductances with frequencies lower than 1 kHz.

### Setups for numerical calculations

For numerical calculations we employed the “Kwant” package^25^, which is based on a tight-binding approximation. The distance between two QPCs *L* = 1.8 μm, QPC width *w* = 400 nm, and Rashba strength *α* = 3.6 × 10^−11^ eVm were adopted to simulate the experiment. The effective mass *m* ^*^ = 0.025 *m*_0_ (where *m*_0_ is the electron mass in a vacuum) and g-factor *g* ^*^ = +8.6 were adopted from previous studies^13^. *E*_F_ was tuned to give the QPC conductance *G*_Q*PC*_ = 1.0*G*_q_. The calculated area was 3.6 μm × 3.0 μm, and was surrounded by absorption walls (except for the QPC passes) and simplified to a square lattice with lattice constant *a* = 25 nm. Impurities were introduced by adding randomly-distributed values to the on-site energy. Typical amplitudes of the impurities were 0.2 eV.

## Electronic supplementary material

Supplementary Information
