# Supplementary material for: Conductance fluctuations in InAs quantum wells possibly driven by Zitterbewegung
Source: Sci Rep. 2017 Aug 11;7:7909. doi: 10.1038/s41598-017-06818-4 (PMC5554240; doi:10.1038/s41598-017-06818-4)
Supplement: Supplementary file 1 — Supplementary Information [file 41598_2017_6818_MOESM1_ESM.pdf]

Conductance fluctuations in InAs quantum wells possibly driven by Zitterbewegung

*Yu Iwasaki, Yoshiaki Hashimoto, Taketomo Nakamura, Shingo Katsumoto
Institute for Solid State Physics, University of Tokyo,
5-1-5 Kashiwanoha, Kashiwa, Chiba 277-8581, Japan
E-mail: you.iwasaki@issp.u-tokyo.ac.jp

Supplementary Note 1: Substrate Characteristics

The substrate of the sample is a (001) semi-insulating InP. The layered structure illustrated in Supplementary Fig.1 (a) was grown by molecular beam epitaxy with lattice matching to the substrate besides an InAs quantum well (QW). Hence the Indium contents in (In,Ga)As and (In,Al)As were 0.53 and 0.52 respectively. The thickness of the InAs QW (4 nm) is far below the critical thickness and pseudomorphic growth is expected. The dopant in n-InAlAs layer is Si and the nominal concentration is 5×10^{18} /cc. Supplementary Figure 1 (b) is the conduction band modulation diagram (black curve) and the envelope of the electron wavefunction (red curve) calculated in a Poisson-Schrödinger sequence. The envelope is localized around the InAs QW and the asymmetry at the two interfaces is expected to produce strong Rashba-type spin-orbit interaction (SOI).

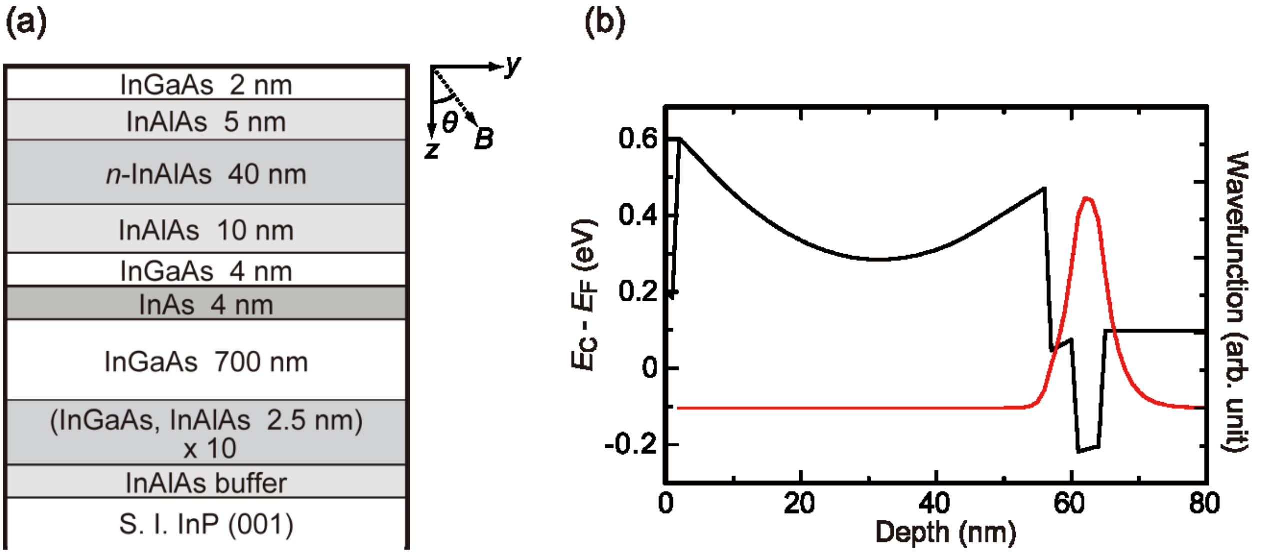

Supplementary Figure 1: Substrate Characteristics: (a) Cross sectional view of the layered structure grown by molecular beam epitaxy. (b) The effective potential (conduction band) diagram of the film as a function of depth from the surface (black) and the envelope of probability amplitude (red) calculated in a Poisson-Schrödinger sequence.

Supplementary Note 2: Shubnikov-de Haas Oscillation

The Rashba strength α was obtained from Shubnikov-de Haas (SdH) resistance oscillation of the two-dimensional electron gas (2DEG) in the InAs QW. Supplementary Fig. 2 (a) shows the magnetoresistance, where SdH oscillation exhibits a node from the beating of two Fermi circles split by Rashba-type SOI [1]. The two frequencies in the oscillation versus inverse of magnetic field are clearly visible in the Fast Fourier Transformation (FFT) shown in Supplementary Fig. 2 (b). The double-peak corresponds to spin-split bands $n^{\pm} = \nu^{\pm} e/h$, where $\nu^{\pm}$ are the peak frequencies. The value of α is obtained from the expression

$$\alpha = \frac{(n^{+} - n^{-})\hbar^{2}}{m^{*}}\sqrt{\pi/[2N_{s} - 2(n^{+} - n^{-})]},$$

where N_s is carrier density. From the data in Supplementary Fig.2, $\alpha = (3.6 \pm 0.5) \times 10^{-11}$ (eV·m) was obtained.

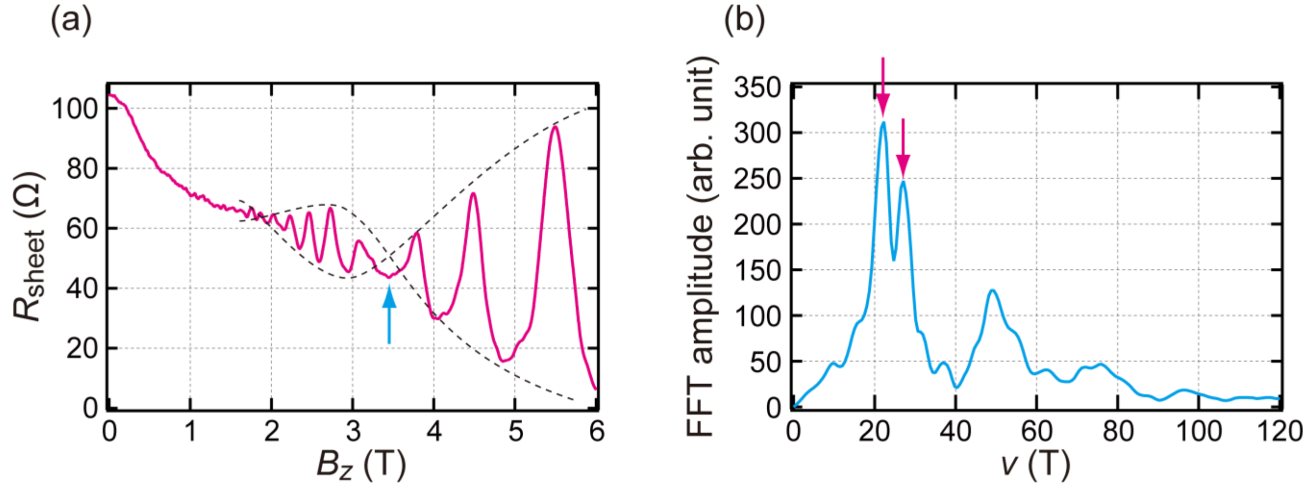

Supplementary Figure 2: Shubnikov-de Haas Oscillation (a) Sheet resistance of the 2DEG as a function of perpendicular magnetic field B_z. The black dotted lines denote the envelope of SdH oscillation with the node indicated by the blue arrow. The temperature is 4.2 K. (b) FFT amplitude spectrum of the SdH oscillation with a double-peak structure as indicated by the arrows.

Supplementary Note 3: Single-QPC Measurement

To clarify the characteristics of quantum point contacts (QPCs), we fabricated a test sample of a single QPC, which has the same trench width and the same open angle with those in the sample used in the experiment described in the paper. “Try and error” tuning of the conductance was not done but instead the gate voltage V_G can be applied to tune the conductance. Supplementary Figure 3 (a) shows the single QPC conductance G_s as a function of V_G. A clear 1.0 G_q plateau is observed, which certifies the conductance quantization at the constriction. As in Supplementary Fig. 3 (b), the conductance fluctuation (CF) in G_s is negligible compared to $G_{1\text{-}5}$, which is through two series QPCs. The result supports the pinball model as the CF appears only when the scattered electrons are collected through a narrow gate.

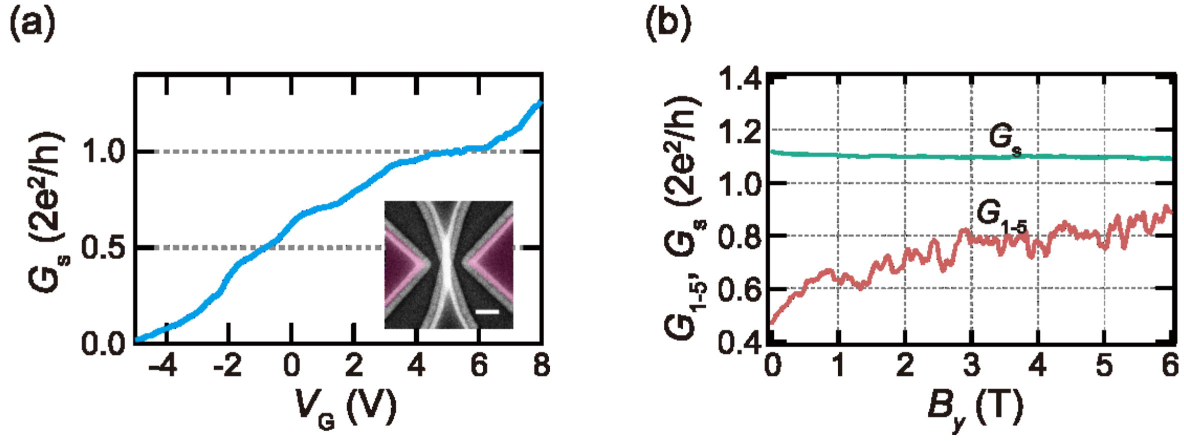

Supplementary Figure 3: Single-QPC Measurement (a) Conductance of a single QPC as a function of gate voltage V_G of the single QPC sample. The inset is an SEM image of the sample with gates shaded in pink false color (T=4.2 K). (b) Comparison of magnetoconductance in G_S and $G_{1\text{-}5}$ (T=100 mK).

Supplementary Note 4: Other Numerical Results

In the numerical calculation, we first confirmed the conductance regularly oscillates as a function of B_y without introducing impurities. Supplementary Figure 4 (a) shows that result, where x-polarized electrons are emitted from the QPC. The oscillation can be interpreted as a change of charge density in the vicinity of the emitter QPC, by comparing it to Fig. 6 (b) and (c). We also conducted conductance calculation as a function of Bx with impurities and y-polarized emission, where G1-5 clearly exhibits CF with lower frequency compared to Fig. 6 (d), agreeing with the experimental results (Fig. 3 (c)).

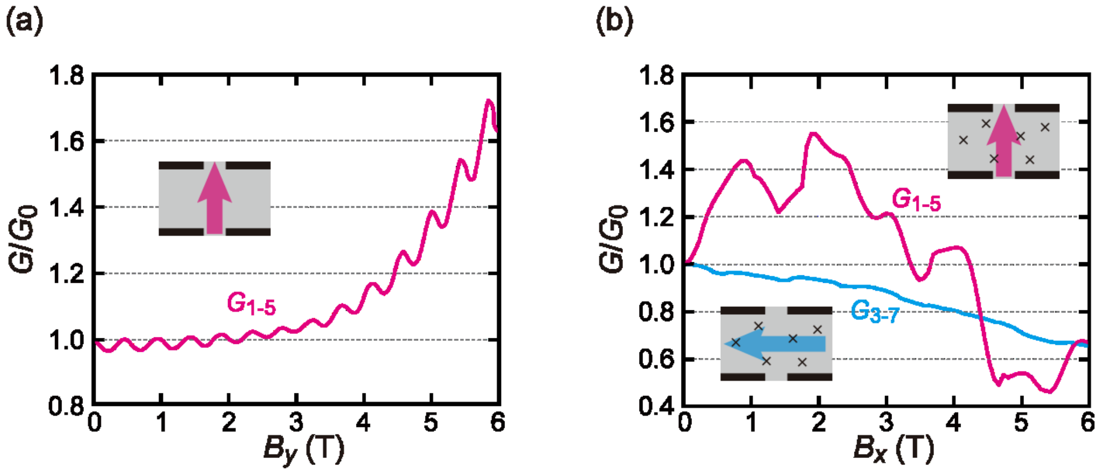

Supplementary Figure 4: Other Numerical Results (a) Calculated $G_{1\text{-}5}$ (with x-polarized emission) as a function of B_y without impurities. (b) Calculated $G_{1\text{-}5}$ (with y-polarized emission) and $G_{3\text{-}7}$ (without polarization) are shown as a function of B_y. Random scattering centers are introduced. Both data of (a) and (b) are normalized by $G_0 = G(\mathrm{B} = 0)$ with illustrations of the probe configurations in the insets.

Supplementary Reference

[1] Grundler, D., Large Rashba Splitting in InAs Quantum Wells due to Electron Wave Function Penetration into the Barrier Layers. *Phys. Rev. Lett.* **84,** 6074-7 (2000)
